# Antibiotics for cancer therapy

**DOI:** 10.18632/oncotarget.3388

**Published:** 2015-02-17

**Authors:** Richard G. Pestell, Albert A. Rizvanov

**Affiliations:** Department of Cancer Biology and Sidney Kimmel Cancer Center, Thomas Jefferson University, Philadelphia, PA, USA and Kazan Federal University, Republic of Tatarstan, Russian Federation

Elegant studies by Lisanti, Sotgia and co-workers demonstrate that when breast cancer cells are grown under conditions that promote 3D-sphere formation, which selects for cancer stem cells, that mitochondrial proteins are dramatically induced. Importantly, these studies also show that antibiotic agents that function as mammalian inhibitors of mitochondrial biogenesis, are selectively toxic to cancer stem cells, when grown as mammospheres [1, 2]. The effects of antibiotics on mitochondrial biogenesis can be explained by the ‘endosymbiotic theory of mitochondrial evolution’, according to which mitochondria directly evolved from bacteria. This is why drugs that target prokaryotic protein synthesis interfere with eukaryotic mitochondrial biogenesis.

The role of mitochondrial metabolism in stem cells and cancer is complex. Stem cells and cancer stem cells have distinct characteristics and the techniques used to analyze these types of cells have often given different results. For example, there is an apparent disparity between transplantation and lineage tracing experiments analyzing mammary gland stem cells.

The metabolism of stem cells differs from normal cells. Undifferentiated embryonic stem cells as well as adult stem cells differ from fully differentiated cells. Stem cells rely mostly on anaerobic metabolism, rather than oxidative phosphorylation. In undifferentiated stems cells, the number of mitochondria are reduced with a low content of mtDNA and a reduced rate of oxygen consumption, as well as a low level of intracellular ATP and reactive oxygen species, consistent with a quiescent state. However, intact mitochondrial function is crucial for the maintenance of stem cells, as evidenced by compromised hematopoietic stem cell function, profound anemia and lymphopenia in mice expressing a mutant form of mitochondrial DNA polymerase-γ that is associated with increased mitochondrial DNA mutations.

In contrast with the reduction in mitochondrial mass in normal stem cells, Lisanti and colleagues demonstrated an increase in mitochondrial protein abundance in breast cancer stem cells, defined by growth as mammospheres. In studies conducted in the early 1980s, tumor carcinoma cells, induced by chemical carcinogens or v-fos correlated with an increase in mitochondrial membrane potential. This increase in mitochondrial membrane potential upon oncogenic transformation was the basis of screening for mitochondrio-toxic drugs that selectively inhibit tumor growth [3]. Oncogenes and tumor suppressors govern mitochondrial biogenesis as either cyclin D1 overexpression, or pRB deletion have similar affects to inhibit mitochondrial biogenesis, via inactivation of NRF1, and thereby MtTFA, to thereby enhance cytosolic glycolysis [4, 5], as observed in the Warburg effect.

In the studies by Sotgia, Lisanti and co-workers, the proteomic analysis conducted on mammary stem cells, identified an increased abundance of mitochondrial proteins in two distinct breast cancer cell lines. The notion that such a change may confer increased sensitivity to antibiotics was examined by treating 3D tumor-spheres and indeed the number of colonies was reduced in 12 cell lines representing 8 different types of cancer, without affecting normal fibroblasts or cells in monolayer. Importantly this is in contrast to the effect of antibiotics, which enhance the renewal of non-malignant stem cells [6]. Further pre-clinical *in vivo* studies are warranted as the local tissue microenvironment creates a metabolic asymmetry and distinct cell types may have different response to these agents. The bioequivalent dose required to block cancer stem cells should be assessed carefully, and the mechanism examined further, given that prior studies suggest that antibiotics can also affect kinase signaling pathways and the secretion of cytokines, including IL-8 [7], which is known to promote cancer stems cell expansion.

The evidence that antibiotics may reduce the viability and clonal expansion of cancer stem cells is of broad importance, as cancer stem cells are increasingly accepted as a distinct cell type that gives rise to therapy resistance, tumor recurrence and distant metastasis.
